# Exploratory Cross-Sectional Associations of Postpartum Maternal Body Mass Index and Infant Anthropometry with Human Milk Hormones and Cytokines

**DOI:** 10.3390/nu18193145

**Published:** 2026-09-24

**Authors:** Mario Daniel Caba-Flores, Gabriela Cruz-Carrillo, Nereida Montes-Castro, Jorge Iván Zurutuza-Lorméndez, Rocío Ortiz-López, Carmen Martínez-Valenzuela, Alberto Camacho-Morales

**Affiliations:** 1 Departamento de Bioquímica y Medicina Molecular, Facultad de Medicina, Universidad Autónoma de Nuevo León, Monterrey 64460, Nuevo León, Mexico; md_caba@hotmail.com (M.D.C.-F.); gabriela.cycr@gmail.com (G.C.-C.); 2 Unidad de Neurometabolismo, Centro de Investigación y Desarrollo en Ciencias de la Salud, Universidad Autónoma de Nuevo León, Monterrey 64460, Nuevo León, Mexico; 3 Departamento de Ciencias de la Salud, Universidad Autónoma de Occidente, Culiacan 80020, Sinaloa, Mexico; nereida.montes@uadeo.mx; 4 Centro de Investigaciones Biomedicas, Universidad Veracruzana, Xalapa 91190, Veracruz, Mexico; jorzurutuza@uv.mx; 5 Escuela de Medicina y Ciencias de la Salud, The Institute for Obesity Research, Tecnológico de Monterrey, Monterrey 64710, Nuevo León, Mexico; rortizl@tec.mx; 6 Unidad de Investigación en Ambiente y Salud, Universidad Autónoma de Occidente, Los Mochis 81216, Sinaloa, Mexico; camava9@gmail.com

**Keywords:** human milk composition, exclusive breastfeeding, infant anthropometry, maternal body mass index, bioactive molecules, milk cytokines, milk hormones, infant sex, birth mode

## Abstract

Background/Objectives: Human milk contains hormonal and immune bioactives that may vary with maternal, infant and perinatal characteristics including maternal body mass index (BMI), infant sex and birth mode. However, evidence linking these factors remains inconsistent. This exploratory study evaluated if maternal, infant and perinatal characteristics were associated with milk composition. Secondarily, hypothesis-generating analyses examined if milk hormones and cytokines were associated with infant anthropometric Z-scores. Methods: This cross-sectional study included 47 healthy term, exclusively breastfed mother–infant dyads with ages ranging from 0.39 to 6.01 months. Milk hormones (ghrelin, leptin, insulin and glucagon) and cytokines (IL-1β, IL-2, IL-4, IL-6, IL-7, MCP-1, IFN-γ and TNF-α) were quantified using multiplex assays and infant anthropometry was evaluated using WHO Z-scores. Adjusted models included infant age, postpartum maternal BMI, infant sex, and birth mode. Analyte-specific complete-case samples ranged from 37 to 44. All *p* values were nominal, and false-discovery-rate-adjusted q values assessed multiplicity. Results: Postpartum maternal BMI was positively associated with milk leptin (β = 0.152; *p* < 0.001; *n* = 39) and cesarean delivery, relative to vaginal delivery, was associated with lower milk glucagon (β = −0.579; *p* < 0.001; *n* = 37). Milk IFN-γ showed nominal exploratory positive associations with weight-for-length Z-score (β = 0.188; *p* = 0.017; *n* = 39) and BMI-for-age Z-score (β = 0.211; *p* = 0.032; *n* = 39). Conclusions: Postpartum maternal BMI and birth mode showed robust adjusted associations with leptin and glucagon, respectively. The cross-sectional design does not establish temporal direction or clinical relevance. The IFN-γ findings require longitudinal evaluation.

## 1. Introduction

Human milk is a dynamic biological fluid containing nutrients and non-nutritive bioactive components, including hormones, cytokines, immune cells and immunoglobulins [1,2]. Its composition may vary according to maternal and infant factors, including maternal body mass index (BMI), infant infection, infant sex, prematurity, lactation stage and birth mode [2,3,4,5,6,7,8].

Overweight and obesity are growing global health problems affecting all age groups, with particularly high rates in Latin America [9,10]. In Mexico, the 2022 National Health and Nutrition Survey reported that 37.3% of children aged 5–11 years and over 72% of adults aged 20 years and older are affected [11]. In parallel, breastfeeding is associated with a lower risk of later overweight and obesity in offspring, potentially through mechanisms related to appetite regulation and healthier gut microbiota [12,13]. International health agencies recommend exclusive breastfeeding for the first 6 months, followed by complementary feeding up to at least 2 years of age [13,14].

Parental adiposity may be relevant to fetal growth and birth size. In a cohort of 899 father–mother–child triplets, higher paternal BMI at conception was associated with birth weight and several measures of intrauterine growth, including biparietal diameter, head circumference and abdominal size in male but not in female offspring, and these associations remained evident after adjustment for maternal BMI, parental age and other clinical, obstetric and metabolic covariates [15]. In a Mendelian randomization study of 30,487 women, a one-SD genetically higher maternal BMI, equivalent to approximately 4 kg/m^2^, was associated with a 55 g higher offspring birth weight, supporting a possible causal relationship between maternal BMI and infant birth weight [16]. Mothers with obesity have also been reported to show differences in bioactive human milk composition, including higher leptin [3,7,17] and insulin levels [7], as well as higher concentrations of IL-2, IL-6, TNF-α and IFN-γ in colostrum [6]. In mature milk, however, lower concentrations of IFN-γ, TNF-α and IL-4 have been reported [6]. Overall, associations between maternal BMI and human milk composition remain inconsistent, with some studies reporting significant changes, minimal alterations or conflicting results [2].

Other maternal factors such as parity have been less explored in relation to nutritive and bioactive human milk components. Infant characteristics including sex and perinatal factors such as birth mode may also be associated with variation in milk composition, with previous studies reporting differences in milk insulin concentrations according to maternal BMI and infant sex [7] and higher TNF-α concentrations after cesarean delivery during early lactation [8]. Understanding whether maternal BMI, parity, infant sex and birth mode are associated with milk composition is important for elucidating the potential relevance of these variations to infant anthropometric status.

Infant anthropometry is commonly evaluated using WHO (World Health Organization) anthropometric Z-scores, including weight-for-age (WAZ), length-for-age (LAZ), head-circumference-for-age (HCZ), weight-for-length (WLZ) and BMI-for-age (BAZ) [18]. Values above +2 SD for WLZ and BAZ indicate overweight, and values above +3 SD indicate obesity [18]. These categories are clinically relevant as early overweight and obesity are associated with increased risk of later overweight, obesity and type 2 diabetes [12,19].

Milk cytokines may contribute to infant development through promoting gastrointestinal tract development [20], potentially improving nutrient absorption, whereas metabolic hormones may contribute through appetite signaling, energy balance and body composition. Accordingly, milk cytokines such as IL-6, TNF-α [7,21,22], IL-1β, IL-2, IL-4 and IFN-γ [22] and metabolic hormones such as leptin, insulin [3,7,21] and ghrelin [3] have been evaluated in relation to infant anthropometry and body composition. However, the evidence remains incompletely characterized, as some studies report positive [19,21], negative or no associations [3,7,21,22] with infant anthropometry and body composition. These heterogeneous findings highlight a clear knowledge gap regarding associations between milk cytokines and metabolic hormones and infant anthropometry during exclusive breastfeeding. It should be acknowledged that lactation stage is also a central source of variation in human milk composition, as hormone and cytokine concentrations may change between transitional and mature milk and throughout lactation [23,24].

Based on the most consistent and direct evidence from human milk studies, the principal hypothesis examined in this exploratory cross-sectional study was that higher postpartum maternal BMI would be associated with higher milk leptin concentrations. Secondarily, hypothesis-generating analyses evaluated associations of postpartum maternal BMI, parity, infant sex, birth mode, and infant anthropometric Z-scores with the full panel of human milk hormones (insulin, ghrelin, leptin, and glucagon) and cytokines (IL-1β, IL-2, IL-4, IL-6, IL-7, MCP-1, IFN-γ, and TNF-α) during exclusive breastfeeding. These markers were selected based on their reported roles in metabolic and immune regulation, their reported associations with obesity and maternal BMI, their potential relevance for infant anthropometry [6,7,16,25] and direct evidence from human milk studies linking these hormones and cytokines with maternal BMI or infant anthropometry.

## 2. Materials and Methods

### 2.1. Study Design, Setting and Ethics

This exploratory cross-sectional study investigated the associations of maternal BMI, infant sex, birth mode, parity and infant anthropometric Z-scores with hormones and cytokines in human milk. The study was conducted at “Centro de Salud Urbano” in Culiacán, Sinaloa, Mexico, between March and July 2022. Ethical approval was obtained from the Bioethics Committee of the Universidad Autónoma de Occidente in Los Mochis, Sinaloa, Mexico (approval no. 03.09/2021). All procedures were performed in accordance with the Declaration of Helsinki. Written informed consent was obtained from all participating mothers before any study procedure.

### 2.2. Participants

Healthy lactating mothers and their infants were recruited during routine pediatric visits. Maternal inclusion criteria were an age of 18–45 years and having had fewer than five previous pregnancies. Infants were eligible if they were aged between 2 weeks and 6 months, born at term (37–42 weeks), had a birth weight between 2500 and 4000 g and were exclusively breastfed, defined as having received only human milk from birth until recruitment, with no previous or current intake of infant formula, water, teas, complementary foods or other liquids except for oral rehydration solution, medications, vitamins or mineral supplements recommended by a pediatrician [26]. Exclusive breastfeeding status was assessed at recruitment via maternal report during the eligibility interview. Mothers were asked if the infant had received any formula, complementary foods or other liquids since birth. Dyads reporting any previous formula exposure were considered non-eligible.

Exclusion criteria included mothers with a history of substance use disorders, immune deficiencies, thyroid disorders, pre-eclampsia, chronic diseases, pre-pregnancy diabetes or gestational diabetes. Infants with congenital abnormalities or metabolic diseases diagnosed at birth or during lactation were also excluded. Mother–infant dyads with acute illness at recruitment, including fever, respiratory or gastrointestinal infection, were also excluded.

The study was exploratory; therefore, no a priori sample size calculation was performed. Of 92 mother–infant dyads initially assessed for eligibility during the recruitment period, 47 met all eligibility criteria, had complete available medical records and were included in the final analysis. Itemized reasons for non-inclusion and detailed clinical or anthropometric characteristics of non-included dyads were not retained in sufficient detail to allow comparison with the analyzed cohort. All participants were volunteers and received no monetary compensation for their participation.

For descriptive and bivariate analyses, mothers were classified by BMI at recruitment as normal weight (18.5–24.9 kg/m^2^) or overweight/obesity (BMI ≥ 25 kg/m^2^). Overweight and obesity were combined to avoid small subgroups with limited representation and to preserve statistical stability. In regression models, maternal BMI was analyzed as a continuous variable.

### 2.3. Maternal and Infant Variables

Maternal variables included age, height, weight, body mass index (BMI), parity (primipara or multipara) and birth mode (cesarean delivery (CD) or vaginal delivery (VD)). Maternal height and weight were measured at recruitment, and current BMI was calculated as weight in kilograms divided by height in square meters. Pre-pregnancy BMI, gestational weight gain, fasting glucose and insulin and lipid profiles were unavailable. Information on maternal place of residence, medication use, dietary supplement or probiotic use and exercise was also unavailable.

Infant variables obtained through medical records included sex (male or female), gestational age, birth length and birth weight. At recruitment, infant length, weight, head circumference and age in months were measured during a pediatric visit by trained health personnel using medical-grade equipment. Infant weight was measured using a Seca Cursa 818 scale (seca GmbH & Co. KG, Hamburg, German). Anthropometric measurements at recruitment were taken in duplicate. When duplicate measurements were discrepant, a third measurement was obtained and the mean of the three measurements was used as the final value for the analyses. Information regarding device calibration schedules, measurement precision records and formal intra or inter-assessor reliability estimates was not available. WHO anthropometric Z-scores (WLZ, WAZ, LAZ, BAZ and HCZ) were calculated using WHO Anthro v 3.2.2 software (World Health Organization, Geneva, Switzerland) according to WHO Child Growth Standards [18,27]. WLZ, WAZ, LAZ and BAZ were calculated at birth and at recruitment, whereas HCZ was calculated only at recruitment. LAZ, WAZ and HCZ were used to describe length, weight and head circumference relative to age, while BAZ and WLZ were used to describe BMI relative to age and weight relative to length, respectively, according to WHO standards [18]. As each infant contributed birth anthropometry and one postnatal anthropometric assessment at variable ages at recruitment, analyses focused on Z-scores at recruitment, and therefore individual longitudinal changes in anthropometry were not calculated. As anthropometric assessment and milk collection occurred on the same day, infant age at recruitment reflected the developmental stage at which biological and anthropometric data were obtained.

### 2.4. Human Milk Collection and Storage

At a single study visit, each mother was provided with a sterile polypropylene tube and instructed to manually express one 10 mL sample from one breast within a standardized morning window (9:00 a.m. to 12:00 p.m.), 2–3 h after the last breastfeeding or pumping session and 5 min after initiating infant feeding to minimize variations in milk bioactives related to collection time [28]. No samples were pooled across collection times or participants. This collection strategy was intended to reduce variations related to collection time, recent feeding and within-feed milk composition. Mothers were instructed to keep samples refrigerated (2–8 °C) and to transport them on the same day to “Centro de Salud Urbano” in Culiacan, Sinaloa, within 4 h using insulated cooler bags and ice packs. Upon arrival, samples were stored at −20 °C until analysis, within a maximum storage window of 3 months. Samples remained frozen until processing and underwent a single freeze–thaw cycle immediately before multiplex analyses. All procedures followed standardized protocols for milk collection, transport and storage to ensure sample integrity.

### 2.5. Milk Hormone and Cytokine Quantification

For human milk analysis, we used a Luminex Multiplex Immunoassay, a technique previously used in human milk studies for hormone and cytokine analysis [23,29,30,31]. Frozen samples were thawed at room temperature, vortexed for 15 s and centrifuged at 605× *g* for 10 min at 4 °C. The fat layer was discarded and 25 µL of the undiluted aqueous phase was used in duplicate for hormone and cytokine quantification. Therefore, measured concentrations correspond to the undiluted aqueous/skim milk fraction rather than whole milk. The hormones insulin, ghrelin, leptin and glucagon were analyzed using the HMHEMAG-34 MILLIPLEX MAP Human Metabolic Hormone Magnetic Bead Panel—Metabolism Multiplex assay kit (Merck Millipore, Burlington, MA, USA). The cytokines IL-1β, IL-2, IL-4, IL-6, IL-7, MCP-1, IFN-γ and TNF-α were analyzed using the HCYTOMAG-60K Human Cytokine Magnetic Bead Panel/Chemokine kit (Merck Millipore, Burlington, MA, USA).

All assays were performed according to the manufacturer instructions in their corresponding hormone or cytokine multiplex panel runs. Samples were arranged by consecutive study ID and were not ordered according to maternal BMI, birth mode, parity, infant sex or infant anthropometric status. No formal within-plate randomization was performed. The operator who performed the multiplex assays received the samples without maternal or infant grouping information and was blinded to these variables during assay processing. Standard curves and manufacturer-provided internal quality controls QC1 and QC2 were processed with samples. For batch acceptance, calibration curves were required to have R^2^ ≥ 0.98, and quality control values were required to fall within manufacturer-defined ranges. Duplicate measurements were evaluated for intra-assay precision, and concentrations below or above the valid quantification range were excluded from the analyses. Quantification was performed using a Luminex^®^ 200^TM^ Multiplexing Instrument (Luminex Corporation, Austin, TX, USA; LX200-XPON3.1).

### 2.6. Statistical Analysis

Maternal and infant characteristics were summarized using means, standard deviations and ranges for continuous variables, and counts and percentages for categorical variables. Cytokine and hormone concentrations were tested for normality using the Shapiro–Wilk test and for homogeneity of variances using the Levene test. Only quantifiable concentrations were included in the primary analyses. Values below the assay limit of quantification or outside the valid standard curve range were treated as missing in these analyses. Bivariate comparisons were performed according to birth mode, maternal BMI group, parity and infant sex. Data were analyzed using unpaired *t*-tests for Gaussian-distributed data and Mann–Whitney U tests for non-Gaussian-distributed data. For bivariate comparisons, false-discovery-rate correction was performed using the Benjamini–Hochberg method within each grouping variable family across the 12 analytes.

Exploratory multiple linear regression models were fitted separately for each milk hormone and cytokine using log(concentration + 1) transformed concentrations as dependent variables. The adjusted model for each analyte included infant age at recruitment and postpartum maternal BMI as continuous covariates and infant sex and birth mode as categorical covariates. This covariate set followed a simple conceptual adjustment rationale: infant sex and birth mode were treated as pre-sampling infant/perinatal characteristics, and infant age at recruitment and postpartum maternal BMI were treated as lactation stage/maternal factors potentially related to milk analyte concentrations.

As none were considered a mediator, they were included simultaneously. Models were interpreted as adjusted associations rather than causal effects. Accordingly, because the analyses were designed to evaluate adjusted associations rather than to estimate a prespecified causal effect, a formal directed acyclic graph (DAG) was not used to define the covariate set. Instead, covariates were selected based on the conceptual rationale described above. Exploratory IFN-γ models additionally included one infant anthropometric Z score (WLZ or BAZ) at a time.

For model assumption assessment, we used residual diagnostics, including the Shapiro–Wilk test for residual normality, the Breusch–Pagan test for heteroscedasticity, variance inflation factors for collinearity, and Cook’s distance to identify potentially influential observations. We also applied false-discovery-rate correction to regression analyses using the Benjamini–Hochberg method within covariate families across the 12 analytes. Nominal *p* values and FDR-adjusted q values are reported.

We performed sensitivity analyses to assess the robustness of the main findings. These included comparison of infant-age distributions across maternal BMI, infant-sex and birth-mode groups, evaluation of nonlinear infant-age effects using a quadratic age term, exclusion of samples collected before 14 days postpartum, exclusion of WHO-flagged anthropometric values and observations with high leverage, and exclusion of protocol deviations related to birth weight or age at recruitment. For IFN-γ and IL-4, Tobit regression models were used as sensitivity analyses for evaluating if left-censored values below the practical limit of quantification modified the interpretation of the main adjusted models. Tobit modes used the same covariate specification as the corresponding primary regression models.

Minimum detectable effects were estimated for a reference adjusted model using the smallest complete case sample size among the main regression analyses (glucagon model, *n* = 37), assuming a two-sided α = 0.05 and 80% power. Estimated MDEs on the log(concentration + 1) scale were 0.104 for infant age, 0.064 for postpartum maternal BMI, 0.454 for infant sex and 0.442 for birth mode.

Given the exploratory nature of the study and the modest sample size, findings were interpreted according to their magnitude, precision, consistency across sensitivity analyses and robustness after FDR correction. Statistical significance was set at *p* ≤ 0.05 for nominal analyses, and q < 0.05 was considered robust to FDR correction. Analyses were performed using GraphPad Prism version 9.0.0, GraphPad Software, San Diego, CA, USA, and R version 3.6.3, R Foundation for Statistical Computing, Vienna, Austria.

## 3. Results

### 3.1. Maternal and Infant Characteristics

Only 47 dyads with complete available records were included. All demographic and anthropometric characteristics of the dyads are summarized in Table 1. Data discrepancies and sensitivity analyses are reported in Section 3.5.

Maternal age ranged from 18 to 36 years (M = 26.51, SD = 4.71) and BMI ranged from 19.09 to 35.56 kg/m^2^ (M = 24.63, SD = 3.39). Based on BMI, 26 mothers were assigned to the normal weight group, and 21 to the overweight/obesity group. Most mothers were primiparous, and vaginal delivery was the most common birth mode. Infant age at recruitment ranged from 0.39 to 6.01 months (M = 2.78, SD = 2.02) and most infants were male. Mean infant anthropometric Z-scores were generally close to WHO reference values, and two individual WLZ values exceeded WHO biologically plausible value thresholds. This is detailed in Section 3.5.

### 3.2. Bivariate Comparisons of Maternal and Infant Characteristics with Human Milk Hormones and Cytokines

Ghrelin, glucagon, insulin, leptin, IL-1β, IL-2, IL-4, IL-6, IL-7, MCP-1, IFN-γ and TNF-α in human milk were analyzed according to birth mode, maternal BMI group, parity and infant sex. After applying false-discovery-rate correction within each grouping variable family, only the association between birth mode and milk glucagon remained robust (*p* < 0.001; q = 0.007; Table 2).

Milk IFN-γ was nominally higher in the overweight/obesity group than in the normal weight group, but did not remain robust after the correction (*p* = 0.006; q = 0.070). Similarly, the association between infant sex and glucagon was also nominally significant, but did not remain robust after correction (*p* = 0.028; q = 0.336). Milk IL-6 was not significantly associated with parity (*p* = 0.148; q = 0.591). The distribution of quantifiable milk hormone and cytokine concentrations is shown in Figure 1.

Consistent with the right-skewed distributions shown in Figure 1, hormone and cytokine concentrations were modeled as log(concentration + 1)-transformed dependent variables in the regression analyses.

### 3.3. Exploratory Multiple Linear Regression Models

Exploratory multiple linear regression models were fitted for each milk hormone and cytokine using log(concentration + 1) transformed concentrations as dependent variables. Each primary model included infant age at recruitment, postpartum maternal BMI, infant sex and birth mode simultaneously. Table 3A presents all coefficients from the main models, including nonsignificant predictors, exact analyte specific *n* values, 95% confidence intervals, nominal *p* values and false-discovery-rate adjusted q values.

In the main models, after simultaneous adjustment for infant age, postpartum maternal BMI, infant sex and birth mode, only two associations remained robust after false-discovery-rate correction. Postpartum maternal BMI showed a positive association with milk leptin (β = 0.152 in log[pg/mL + 1] per kg/m^2^; 95% CI 0.068 to 0.236; *p* < 0.001; q = 0.010; *n* = 39), and cesarean delivery, relative to vaginal delivery, showed a negative association with milk glucagon (β = −0.579 in log[pg/mL + 1] 95% CI −0.890 to −0.268; *p* < 0.001; q = 0.007; *n* = 37).

Although milk glucagon differed nominally by infant sex in bivariate analyses, infant sex was not associated with glucagon in the adjusted model that included birth mode simultaneously (β = −0.156; 95% CI −0.476 to 0.165; *p* = 0.329; q = 0.954). Infant age showed a nominal positive association with IFN-γ (β = 0.149; 95% CI 0.002 to 0.296; *p* = 0.047), but this association did not remain robust after correction (q = 0.363). Postpartum maternal BMI was not statistically associated with IFN-γ in the adjusted model (β = 0.070; 95% CI −0.015 to 0.156; *p* = 0.104; q = 0.450).

To evaluate whether infant anthropometric status was associated with milk IFN-γ, infant WLZ and BAZ were added separately to the adjusted IFN-γ model, one anthropometric Z-score at a time. WLZ showed a nominal positive association with IFN-γ (β = 0.188; 95% CI 0.036 to 0.341; *p* = 0.017; *n* = 39), and BAZ also showed a nominal positive association with IFN-γ (β = 0.211; 95% CI 0.020 to 0.401; *p* = 0.032; *n* = 39). Because WLZ and BAZ are related anthropometric indices, these were interpreted together rather than as independent findings.

Model diagnostics are summarized in Table 3B. The Breusch–Pagan test indicated heteroscedasticity in the glucagon model, and Shapiro–Wilk tests indicated deviations from residual normality in the IFN-γ, IL-2, IL-4 and MCP-1 models. Variance inflation factors were low in all models (<1.3), indicating no relevant collinearity among infant age, postpartum maternal BMI, infant sex and birth mode. Observations with high Cook’s distance were evaluated in sensitivity analyses.

Selected associations involving continuous variables are shown in Figure 2. Scatterplots are used to display the relationships of postpartum maternal BMI with milk leptin and IFN-γ and milk IFN-γ with infant WLZ and BAZ. Each panel shows the fitted regression line, 95% confidence band, regression estimate and influential observations identified using Cook’s distance > 4/*n*.

### 3.4. Sensitivity Analyses for Lactation Stage

As infant age at recruitment ranged from 0.39 to 6.01 months, we evaluated if lactation stage differed across the main grouping variables and whether nonlinear age effects influenced the main regression models. Infant age did not differ by maternal BMI group (median 2.29 vs. 2.20 months in normal-weight vs. overweight/obesity groups; *p* = 0.923), infant sex (2.17 vs. 2.37 months in female vs. male infants; *p* = 0.840), or birth mode (2.15 vs. 2.30 months in cesarean vs. vaginal delivery; *p* = 0.605).

To assess potential nonlinear lactation-stage effects, a quadratic infant-age term was added to each of the 12 main regression models. No analyte showed evidence of a nonlinear association with infant age (all *p* > 0.10), and infant age was therefore retained as a linear covariate in the main models.

Finally, sensitivity analyses excluding the five dyads with samples collected before 14 days postpartum showed that the two robust associations from the main regression models remained statistically significant: birth mode with glucagon (β = −0.686; *p* < 0.001; *n* = 34) and postpartum maternal BMI with leptin (β = 0.172; *p* = 0.001; *n* = 34). The exploratory IFN-γ associations with WLZ and BAZ also remained nominally significant after this exclusion (WLZ: β = 0.201; *p* = 0.011; *n* = 34; BAZ: β = 0.219; *p* = 0.015; *n* = 34). Given the modest sample size, the results were interpreted as exploratory adjusted associations.

The IFN-γ associations with infant WLZ and BAZ were also displayed after stratification by infant-age tertiles for evaluating if these associations were restricted to a single lactation-stage subgroup. As shown in Figure 3, the positive direction of these associations can be observed across infant-age tertiles. However, this stratified visualization should be interpreted as exploratory due to the small number of observations per stratum.

### 3.5. Sensitivity Analyses for Eligibility Deviations, Anthropometric Plausibility and Influential Observations

Eligibility and anthropometric data were rechecked to identify discrepancies in birth weight, infant age at recruitment and biologically plausible anthropometric values. One dyad had a birth weight of 4.30 kg, which was higher than the eligibility criteria of 4.0 kg. Four dyads were recruited at 183 days, corresponding to 6.01 months, which was slightly above the prespecified upper age limit of 6 months (182 days). WHO biologically plausible-value thresholds identified 2 dyads with implausible WLZ values: one at birth (WLZ = −5.12) and the other at recruitment (WLZ = 5.53). No other anthropometric Z-score at birth or recruitment exceeded WHO plausibility thresholds.

Sensitivity analyses were performed after excluding the combined set of dyads with eligibility deviations or WHO-flagged WLZ values. This exclusion involved six different dyads, as one met both the age criteria and the WHO-flagged WLZ criteria. The adjusted association between birth mode and glucagon remained statistically significant after this exclusion (β = −0.664; *p* < 0.001; *n* = 31), as well as the adjusted association between postpartum maternal BMI and leptin (β = 0.136; *p* = 0.004; *n* = 33).

Additional sensitivity analyses excluded WHO-flagged WLZ values and model-specific high-leverage observations identified using Cook’s distance > 4/*n*. With this approach, the adjusted association between birth mode and glucagon remained statistically significant (β = −0.505; *p* = 0.001; *n* = 35), as well as the adjusted association between postpartum maternal BMI and leptin (β = 0.148; *p* = 0.001; *n* = 38). The IFN-γ–BAZ association also remained statistically significant (β = 0.212; *p* = 0.026; *n* = 34), but the IFN-γ–WLZ association was attenuated and did not reach conventional statistical significance (β = 0.139; *p* = 0.056; *n* = 35). Therefore, the IFN-γ–WLZ finding was interpreted with greater caution.

### 3.6. Sensitivity Analyses for Left-Censored Measurements

As left-censored values were present for IFN-γ and IL-4, Tobit regression models were fitted as sensitivity analyses. This was performed using the same covariate specification as the primary adjusted models. The practical limit of quantification was estimated at 3.0 pg/mL for IFN-γ and at 10.0 pg/mL for IL-4, both with one censored value. The Tobit analyses did not change the interpretation of the primary FDR based findings. Thus, left censoring was not considered to alter the main results in the adjusted models.

## 4. Discussion

Human milk composition is dynamic, yet the maternal, infant and perinatal factors associated with variations in milk hormones and cytokines remain incompletely characterized. In this exploratory cross-sectional study of healthy, term, exclusively breastfed mother–infant dyads, postpartum maternal BMI was positively associated with milk leptin, and cesarean delivery was associated with lower milk glucagon. Secondary analyses also showed exploratory positive associations between milk IFN-γ and infant WLZ and BAZ, but these findings require cautious interpretation given the exploratory and cross-sectional nature of the study.

Among the evaluated hormones and cytokines, milk leptin showed the clearest association with maternal BMI. Leptin is a metabolic hormone involved in appetite regulation, energy expenditure, and adiposity, and its concentration in human milk has repeatedly been associated with maternal BMI and body fat [3,7,32]. In our study, leptin did not differ between the two BMI groups, but it increased in relation to maternal BMI when BMI was analyzed as a continuous variable. This may indicate that categorizing BMI into groups may reduce sensitivity in small exploratory samples, while analyzing BMI as a continuous variable may better reflect gradual variation in milk leptin concentrations.

Importantly, maternal BMI was measured at recruitment and therefore represents BMI during lactation rather than pre-pregnancy BMI [3,33]. As both pre pregnancy BMI and gestational weight gain were unavailable, their contribution to the association with milk leptin could not be evaluated. The association between postpartum BMI and milk leptin was maintained after excluding samples collected before 14 days postpartum. While this suggests that the association was not restricted to the earliest samples in the cohort, postpartum timing may still have influenced maternal BMI and milk leptin concentrations.

Sample processing should be considered when interpreting this finding, as the fat layer was removed from milk samples, and therefore the measurements reflect leptin in the aqueous/skimmed milk fraction rather than total leptin in whole milk. Previous studies have detected leptin in both whole and skimmed human milk, although higher concentrations have been reported in whole milk, probably because the skimming process excludes fat and cellular fractions that may contain additional leptin [34,35]. Nevertheless, associations between maternal adiposity and milk leptin have been observed in studies analyzing both skimmed and whole milk preparations [32,36]. Thus, our findings are consistent with an association between postpartum BMI and leptin levels in the aqueous milk fraction, but this should not be interpreted as an estimate of total milk leptin concentration or as a measure of infant leptin exposure. Future studies should measure leptin in whole milk, include repeated milk sampling, and estimate milk intake, as analyte concentration alone does not represent the amount received by the infant [37]. The same limitation applies to the other analytes, as their concentrations were also measured only in skimmed milk. However, the effect of removing the fat layer on each analyte was not evaluated.

Glucagon is a metabolic hormone and a key regulator of glucose homeostasis [38], but it has received limited attention in human milk research compared with other metabolic hormones such as leptin, insulin, adiponectin or ghrelin [39]. In our study, cesarean delivery was associated with lower milk glucagon concentrations, and this association remained after accounting for maternal and infant characteristics and in the principal sensitivity analyses. Glucagon was also higher among mothers of female infants in the initial comparison, but the sex difference was no longer evident after birth mode and other maternal and infant characteristics were considered. The underrepresentation of female infants (16 female versus 31 male infants) may have limited the precision of this comparison; therefore, this bivariate difference should not be considered as evidence of a sex-related effect. Such differences in milk glucagon should be examined in larger, sex-balanced cohorts.

The literature suggests that delivery mode may be associated with differences in nutritional, hormonal, immunological and microbial milk components [40]. However, these differences are heterogeneous and are generally more evident in colostrum and transitional milk than in mature milk [40]. In our study, the glucagon association persisted even after excluding samples collected before 14 days postpartum, suggesting that it was not limited to the earliest lactation period in this cohort. However, factors related to birth mode including labor exposure, elective versus emergency cesarean delivery, antibiotic prophylaxis and anesthesia may also contribute to variations in milk composition [40]. As information on these factors was not available, the observed associations cannot be attributed specifically to cesarean delivery and may reflect unmeasured obstetric or perinatal characteristics associated with delivery mode. Larger longitudinal studies with detailed obstetric information and repeated milk sampling are needed to evaluate if this finding can be replicated.

The IFN-γ findings require cautious interpretation. Although milk IFN-γ was higher in the overweight/obesity group in bivariate comparisons, this difference did not remain significant after correcting for multiple comparisons. In addition, postpartum maternal BMI was not associated with IFN-γ in the adjusted analysis. Milk IFN-γ showed modest, exploratory positive associations with current WLZ and BAZ. However, as milk and anthropometry were assessed at the same visit and no anthropometric trajectory was measured, these findings do not represent infant growth or establish directionality. Thus, these associations may reflect shared maternal or infant factors, age-related variation or differences in milk IFN-γ associated with the infant current anthropometric status, rather than an influence of this analyte on infant growth. Although the WLZ finding weakened in sensitivity analyses and the BAZ finding was less affected, the magnitude and stability of both associations remain uncertain. These findings are consistent with previous reports showing that associations between individual human milk bioactives and infant anthropometry remain inconsistent [39].

Taken together, the most consistent associations observed in this exploratory study involved leptin and glucagon. These findings reinforce the importance of distinguishing findings supported across adjusted and sensitivity analyses from isolated nominal results and of considering human milk as an integrated biological system in which its composition may vary in relation to infant, maternal and perinatal factors. Although infant age was included in all adjusted models, it is only a proxy for lactation stage and may not fully capture analyte-specific changes in milk composition throughout lactation.

### Strengths and Limitations

This study has several strengths, including the inclusion of healthy term exclusively breastfed dyads, same-day milk collection and anthropometric assessment, standardized milk collection and transport procedures, and simultaneous quantification of multiple metabolic hormones and cytokines. Additional strengths include blinding of the laboratory operator to maternal and infant characteristics, duplicate analyte measurements with internal quality controls and the use of FDR correction and sensitivity analyses to evaluate the consistency of the main findings.

Our study also has limitations. First, the sample was restricted to healthy term dyads, so our findings should not be generalized to preterm or medically complex populations. In addition, because detailed reasons for non-inclusion and characteristics of non-included dyads were unavailable, the possibility of selection bias cannot be excluded. Second, despite the standardized collection timing and procedure, the use of one sample per participant could not fully capture within-feed, diurnal or longitudinal variation in milk composition. Third, the cross-sectional design limits causal inference and precludes assessment of temporal changes in milk composition or longitudinal infant anthropometric trajectories. Fourth, milk samples were skimmed before multiplex assaying, so measured concentrations reflect the aqueous/skimmed milk fraction rather than whole milk. In addition, not all analytes were quantifiable in all samples, resulting in analyte-specific complete-case analyses and reduced sample sizes for some models. Complete-analyte-specific validation including duplicate CVs, recovery, dilution linearity and human milk matrix effect assessments was also not available. Therefore, the measured analyte concentrations and magnitude of the observed associations should be interpreted with appropriate caution. The modest sample size, limited number of exploratory comparisons and underrepresentation of female infants may have reduced statistical power and precision. Residual non-normality or heteroscedasticity was also present in some regression models, despite outcome transformation. Although infant age was included in all main models and sensitivity analyses addressed lactation stage and WHO-flagged anthropometric values, residual confounding by lactation stage variability and anthropometric measurement error cannot be excluded. Fifth, total milk intake and infant intake of milk analytes were not measured, and maternal serum was not analyzed; therefore, serum–milk correlations could not be explored. Pre-pregnancy BMI, gestational weight gain, maternal diet and environmental exposures were also unavailable, limiting interpretation of maternal metabolic or contextual influences. Maternal place of residence, medication use, dietary supplement or probiotic use and physical activity were also unavailable and may have contributed to residual confounding. These limitations should be recognized when interpreting our exploratory findings.

## 5. Conclusions

In this exploratory study, the most consistent associations with selected human milk bioactives involved postpartum maternal BMI and birth mode. Postpartum maternal BMI was positively associated with milk leptin, whereas cesarean delivery was associated with lower milk glucagon. Milk IFN-γ also showed exploratory associations with current infant WLZ and BAZ; however, these findings cannot establish directionality or represent changes in infant growth. Additional longitudinal studies with repeated milk sampling and anthropometric assessments are needed to evaluate these relationships. Importantly, these findings should not be interpreted as indicating that maternal BMI represents a barrier to breastfeeding.

## Figures and Tables

**Figure 1:**
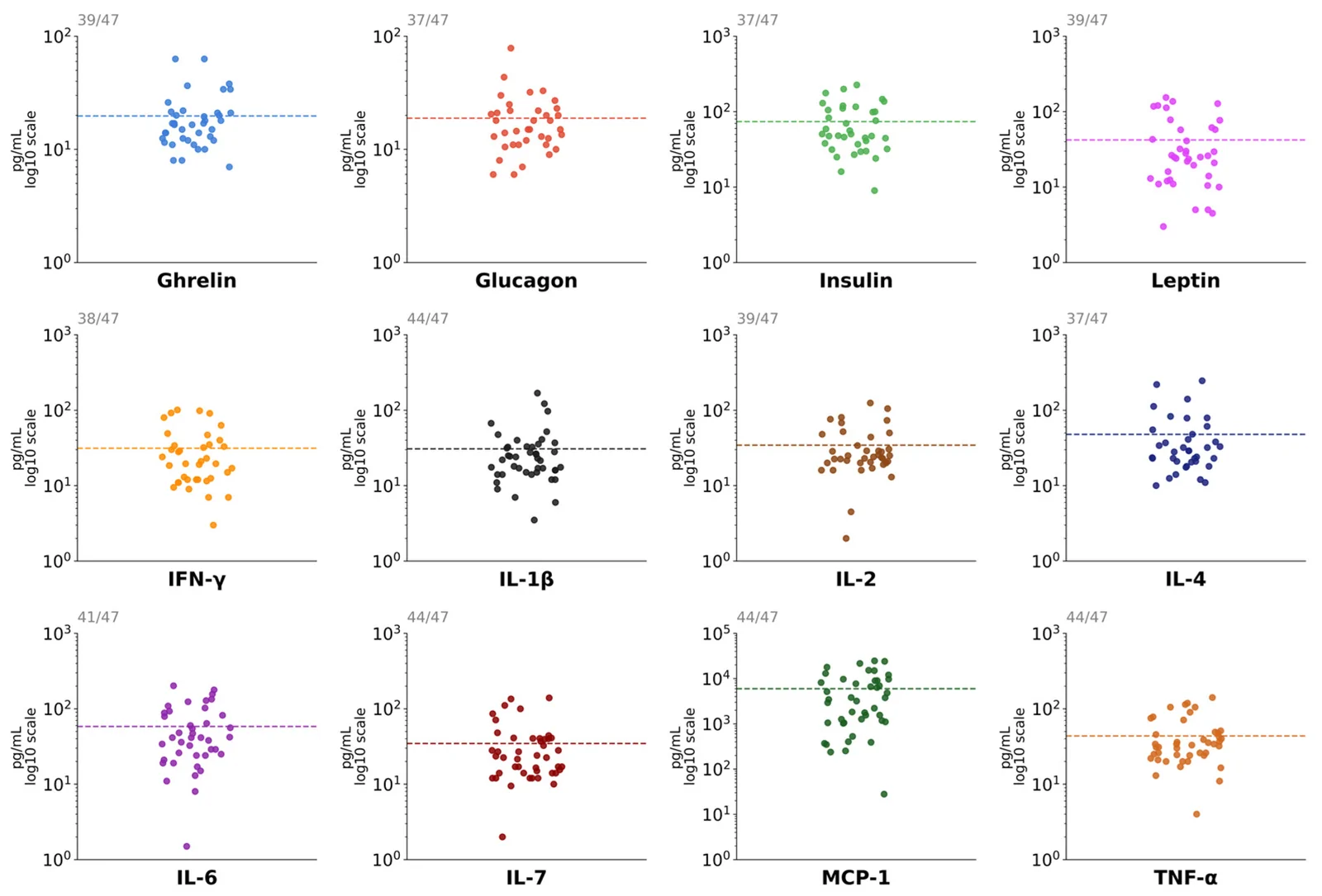
Distribution of human milk hormone and cytokine concentrations. Scatter dot plots show individual quantifiable concentrations for each analyte. Concentrations are expressed in pg/mL. Dashed horizontal lines indicate the median. Colors are used only to visually distinguish the analytes. Y-axes are shown on a log10 scale, and axis limits vary by analyte to accommodate the observed concentration ranges. Numbers above each panel indicate the number of quantifiable samples from the total cohort (*n*/47).

**Figure 2:**
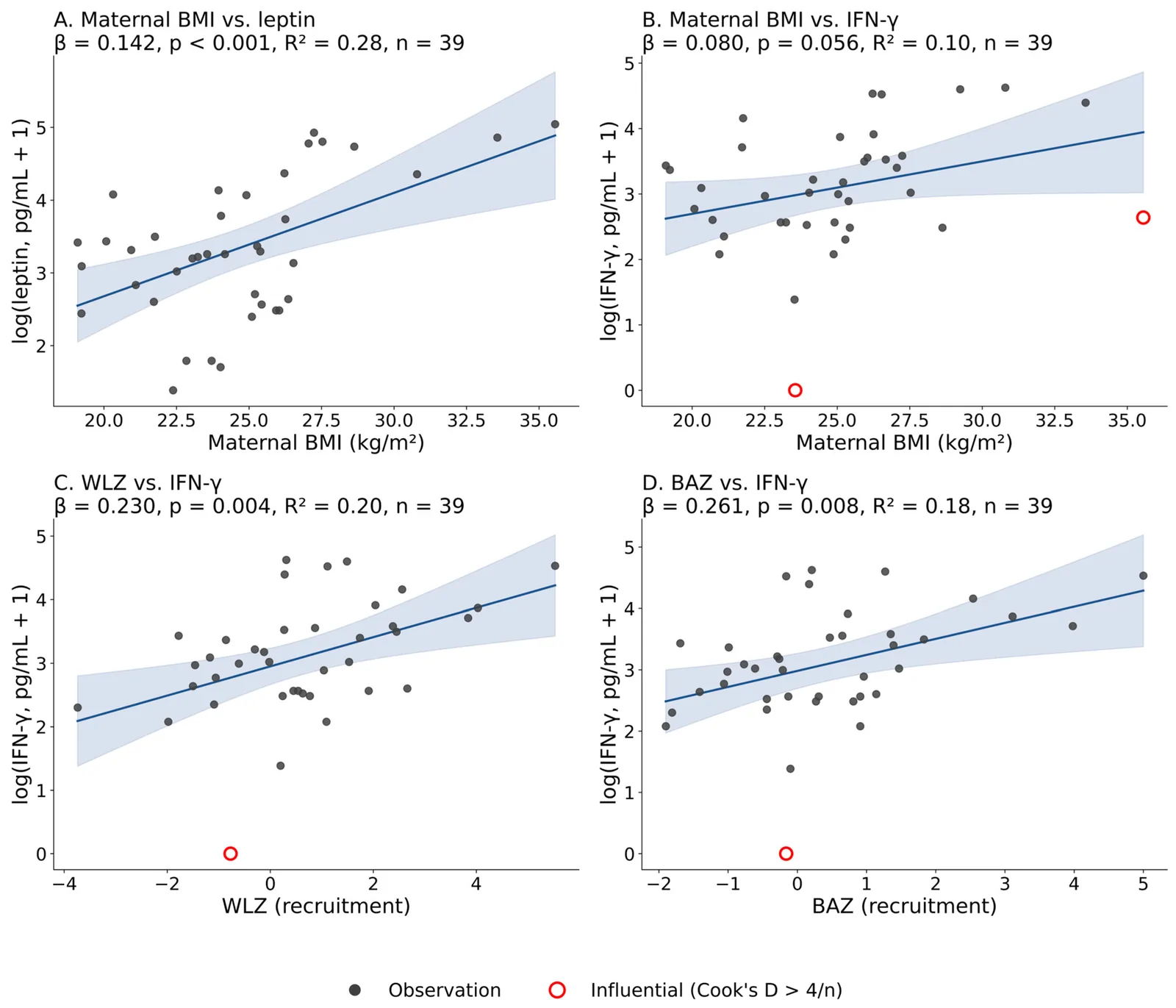
Scatterplots of the principal associations involving continuous variables. Scatterplots show bivariate associations between (**A**) postpartum maternal BMI and milk leptin; (**B**) postpartum maternal BMI and milk IFN-γ; (**C**) infant WLZ and milk IFN-γ; and (**D**) infant BAZ and milk IFN-γ. Hormone and cytokine concentrations are shown as log(concentration + 1) transformed values. Solid blue lines indicate fitted linear regression lines, blue shaded bands indicate 95% confidence bands, gray points represent individual observations, and red open circles indicate influential observations identified using Cook’s distance > 4/*n*. β estimates, *p* values, R^2^ values and analyte-specific *n* values are displayed within each panel. BMI, body mass index; IFN-γ, interferon gamma; WLZ, weight-for-length Z-score; BAZ, BMI-for-age Z-score.

**Figure 3:**
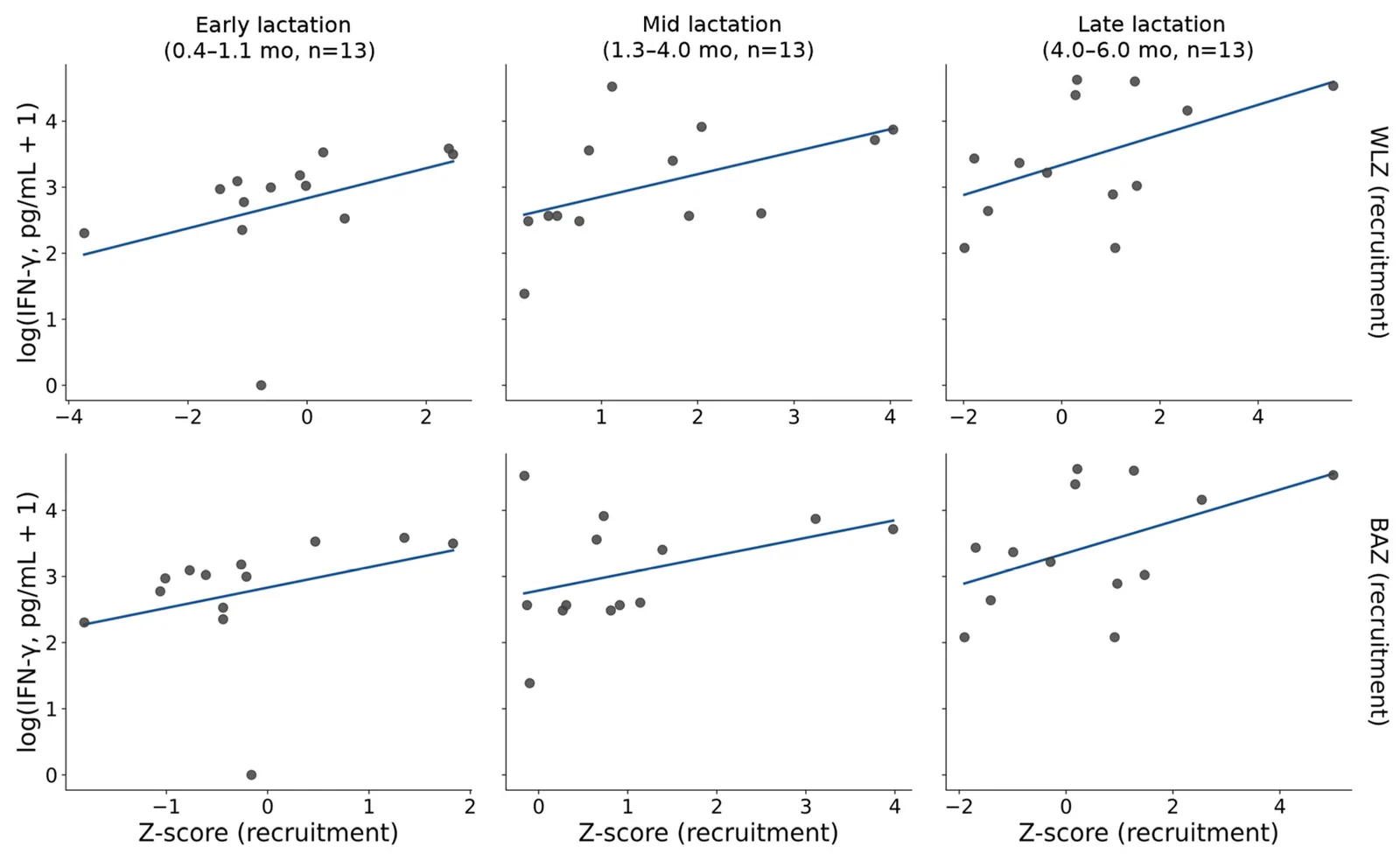
Associations of milk IFN-γ with infant WLZ and BAZ stratified by infant age tertiles. Scatterplots show the relationships of milk IFN-γ with the anthropometric indicators weight-for-length Z-score (WLZ) and BMI-for-age Z-score (BAZ) at recruitment, stratified by infant-age tertiles. IFN-γ concentrations are shown as log(concentration + 1)-transformed values. Gray dots represent individual observations, and blue solid lines indicate the fitted linear regression line within each corresponding infant-age stratum. Confidence bands are due to the small number of observations per panel. The stratified visualization was used to assess if the direction of the IFN-γ associations with WLZ and BAZ was restricted to a single infant-age subgroup. IFN-γ, interferon gamma; WLZ, weight-for-length Z-score; BAZ, BMI-for-age Z-score.

**Table 1:** Maternal and infant characteristics of the study cohort.

Characteristic	Time Point Catery	Total Cohort (*n* = 47)		Male Infants (*n* = 31)		Female Infants (*n* = 16)	
		Mean (SD) or *n* (%)	Range	Mean (SD)	Range	Mean (SD)	Range
Maternal characteristics							
Age, years	Recruitment	26.51 (4.71)	18–36				
Body mass index, kg/m^2^	Recruitment	24.63 (3.39)	19.09–35.56				
BMI group, *n* (%)	Normal weight (18.5–24.9 kg/m^2^)	26 (55.3%)					
BMI group, *n* (%)	Overweight/obesity (≥25 kg/m^2^)	21 (44.7%)					
Parity, *n* (%)	Primiparous	37 (78.7%)					
Parity, *n* (%)	Multiparous	10 (21.3%)					
Birth mode, *n* (%)	Vaginal delivery	27 (57.4%)					
Birth mode, *n* (%)	Cesarean delivery	20 (42.6%)					
Infant characteristics							
Infant sex, *n* (%)	Male	31 (66.0%)					
Infant sex, *n* (%)	Female	16 (34.0%)					
Gestational age, weeks	At birth	38.91 (1.25)	37–41				
Infant age, months	Recruitment	2.78 (2.02)	0.39–6.01				
Infant anthropometry							
Weight, kg	At birth	3.43 (0.39)	2.69–4.30	3.54 (0.39)	2.80–4.30	3.23 (0.31)	2.69–4.00
Weight, kg	Recruitment	5.79 (1.96)	3.10–10.40	6.00 (1.97)	3.32–10.40	5.38 (1.93)	3.10–8.75
Length, cm	At birth	50.62 (2.28)	44–56	51.32 (1.78)	48–56	49.25 (2.57)	44–54
Length, cm	Recruitment	58.56 (6.61)	50.00–71.00	59.31 (6.66)	51.00–71.00	57.13 (6.48)	50.00–68.50
Head circumference, cm	At birth	Not determined		Not determined		Not determined	
Head circumference, cm	Recruitment	39.92 (3.59)	34.00–47.00	40.32 (3.67)	34.00–47.00	38.96 (3.32)	35.00–44.50
WHO anthropometric Z-scores							
WLZ	At birth	−0.30 (1.60)	−5.12 to 2.51	−0.34 (1.68)	−5.12 to 2.00	−0.24 (1.47)	−3.63 to 2.51
WLZ	Recruitment	0.46 (1.77)	−3.74 to 5.53	0.48 (1.97)	−3.74 to 5.53	0.42 (1.33)	−3.04 to 2.56
LAZ	At birth	0.51 (1.15)	−2.76 to 3.23	0.75 (0.95)	−1.00 to 3.23	0.05 (1.38)	−2.76 to 2.60
LAZ	Recruitment	−0.33 (1.51)	−4.04 to 2.13	−0.27 (1.65)	−4.04 to 2.13	−0.46 (1.24)	−2.49 to 1.69
WAZ	At birth	0.23 (0.75)	−1.25 to 1.79	0.36 (0.77)	−1.18 to 1.79	−0.02 (0.66)	−1.25 to 1.57
WAZ	Recruitment	0.03 (1.01)	−1.70 to 3.67	0.11 (1.03)	−1.70 to 3.67	−0.13 (1.00)	−1.53 to 1.64
BAZ	At birth	−0.05 (1.27)	−3.25 to 2.63	−0.06 (1.29)	−3.25 to 1.80	−0.03 (1.29)	−2.63 to 2.63
BAZ	Recruitment	0.31 (1.43)	−2.04 to 5.00	0.39 (1.59)	−1.90 to 5.00	0.16 (1.07)	−2.04 to 2.54
HCZ	At birth	Not determined		Not determined		Not determined	
HCZ	Recruitment	0.51 (1.21)	−1.66 to 3.02	0.56 (1.31)	−1.66 to 3.02	0.39 (0.95)	−1.30 to 1.78

Data are presented as the mean (standard deviation) and range for continuous variables and *n* (%) for categorical variables. Sex-stratified values are presented descriptively, and no statistical comparisons by infant sex were performed. BMI, body mass index; WLZ, weight-for-length Z-score; LAZ, length-for-age Z-score; WAZ, weight-for-age Z-score; BAZ, BMI-for-age Z-score; HCZ, head-circumference-for-age Z-score. Head circumference and HCZ at birth were not available.

**Table 2:** Bivariate comparisons of human milk hormone and cytokine concentrations according to maternal, infant, and perinatal characteristics.

Panel A. Birth mode (A = Vaginal delivery; B = cesarean delivery)							
Analyte	*n* (A/B)	Median (A/B) pg/mL	HL	95% CI (HL)	r	*p*	q
Ghrelin	22/17	17.00/15.00	1.50	−3.0 to 6.0	−0.142	0.461	0.953
Glucagon	21/16	20.50/11.50	8.50	4.0 to 14.0	−0.670	<0.001	0.007
Insulin	22/15	47.75/59.00	−8.50	−32.0 to 21.0	0.121	0.546	0.953
Leptin	22/17	25.25/25.00	1.25	−27.0 to 14.5	−0.024	0.910	0.953
IFN-γ	25/14	19.50/22.50	0.75	−12.0 to 12.5	−0.014	0.953	0.953
IL-1β	26/18	17.75/24.50	−2.75	−11.0 to 5.5	0.083	0.650	0.953
IL-2	25/14	23.00/26.75	−1.50	−8.5 to 11.5	0.049	0.815	0.953
IL-4	25/13	29.00/23.50	−0.50	−18.0 to 11.5	0.028	0.902	0.953
IL-6	25/16	42.00/39.75	10.00	−12.0 to 40.5	−0.183	0.336	0.953
IL-7	25/19	27.00/21.50	2.00	−6.5 to 14.0	−0.084	0.644	0.953
MCP-1	25/19	3445.00/3186.00	−387.50	−2771.5 to 2591.5	0.082	0.653	0.953
TNF-α	26/18	34.00/30.50	4.50	−6.0 to 17.0	−0.147	0.417	0.953
Panel B. Maternal BMI group (A = Normal weight; B = Overweight/Obesity)							
Analyte	*n* (A/B)	Median (A/B) pg/mL	HL	95% CI (HL)	r	*p*	q
Ghrelin	21/18	17.00/15.25	1.50	−3.0 to 6.0	−0.132	0.490	0.534
Glucagon	21/16	14.50/16.50	−3.00	−8.0 to 2.5	0.217	0.269	0.359
Insulin	21/16	70.00/42.75	19.25	−5.0 to 54.5	−0.321	0.101	0.280
Leptin	21/18	24.00/34.50	−17.00	−72.0 to 1.5	0.333	0.078	0.280
IFN-γ	19/20	12.50/32.50	−16.25	−30.5 to −5.0	0.518	0.006	0.071
IL-1β	24/20	17.75/29.50	−7.75	−18.0 to 1.5	0.279	0.117	0.280
IL-2	19/20	22.50/26.50	−5.00	−19.5 to 1.5	0.300	0.112	0.280
IL-4	19/19	23.00/29.00	−7.00	−25.0 to 5.5	0.219	0.255	0.359
IL-6	23/18	42.00/38.75	−3.50	−27.0 to 18.5	0.053	0.783	0.783
IL-7	25/19	17.00/27.00	−5.50	−16.0 to 2.0	0.244	0.172	0.345
MCP-1	24/20	2829.75/4171.00	−722.25	−5128.0 to 1021.0	0.167	0.352	0.422
TNF-α	24/20	31.50/35.00	−6.00	−18.0 to 5.0	0.206	0.248	0.359
Panel C. Parity (A = Multiparous; B = Primiparous)							
Analyte	*n* (A/B)	Median (A/B) pg/mL	HL	95% CI (HL)	r	*p*	q
Ghrelin	7/32	16.50/16.75	0.00	−7.0 to 8.0	0.009	0.985	0.985
Glucagon	7/30	20.00/14.75	5.00	−4.0 to 12.5	−0.267	0.286	0.617
Insulin	6/31	58.75/50.50	1.75	−37.0 to 52.0	−0.038	0.902	0.985
Leptin	5/34	78.00/24.50	42.50	1.0 to 101.5	−0.553	0.051	0.591
IFN-γ	8/31	21.25/19.50	2.00	−13.0 to 16.0	−0.109	0.651	0.977
IL-1β	9/35	17.00/24.00	−5.50	−16.0 to 5.0	0.232	0.295	0.617
IL-2	8/31	25.25/24.00	−1.00	−16.5 to 9.5	0.036	0.889	0.985
IL-4	8/30	30.00/26.25	6.25	−9.0 to 32.0	−0.200	0.400	0.685
IL-6	8/33	44.75/38.00	19.00	−8.0 to 86.5	−0.337	0.148	0.591
IL-7	9/35	27.00/22.50	0.00	−13.0 to 13.0	−0.016	0.954	0.985
MCP-1	10/34	7544.25/3063.25	2324.75	−803.5 to 8793.5	−0.344	0.104	0.591
TNF-α	9/35	45.50/32.00	8.50	−8.0 to 30.0	−0.225	0.308	0.617
Panel D. Infant Sex (A = Female; B = Male)							
Analyte	*n* (A/B)	Median (A/B) pg/mL	HL	95% CI (HL)	r	*p*	q
Ghrelin	14/25	15.00/17.00	0.00	−5.5 to 4.0	0.009	0.977	0.977
Glucagon	13/24	20.00/13.00	5.25	1.0 to 10.0	−0.446	0.028	0.336
Insulin	16/21	49.00/50.50	−6.25	−29.5 to 22.0	0.089	0.657	0.977
Leptin	15/24	26.00/25.00	1.50	−14.0 to 18.5	−0.058	0.773	0.977
IFN-γ	13/26	17.00/20.25	2.00	−10.0 to 14.0	−0.041	0.846	0.977
IL-1β	14/30	17.50/24.25	−4.00	−11.5 to 6.0	0.093	0.632	0.977
IL-2	13/26	23.00/26.75	−2.00	−11.5 to 5.0	0.101	0.623	0.977
IL-4	12/26	23.00/32.50	−10.00	−32.0 to 1.5	0.362	0.078	0.470
IL-6	13/28	32.50/47.50	−14.75	−39.0 to 7.0	0.255	0.197	0.789
IL-7	14/30	19.75/25.25	0.00	−13.0 to 10.0	0.029	0.890	0.977
MCP-1	14/30	4171.00/3192.75	155.00	−2336.5 to 4785.5	−0.052	0.791	0.977
TNF-α	14/30	33.50/33.00	0.00	−12.0 to 12.0	−0.014	0.950	0.977

Table is divided into four panels according to the grouping variable analyzed: Panel A, birth mode; Panel B, maternal BMI group; Panel C, parity; and Panel D, infant sex. *n* values indicate the number of quantifiable observations included in each comparison group, and A and B indicate the comparison groups in the order defined in each panel heading. Concentrations are expressed as pg/mL and presented as medians. HL, Hodges–Lehmann difference between groups (A − B), with its 95% confidence interval calculated using the Hollander–Wolfe normal approximation; r, rank-biserial correlation used as the effect-size estimate; *p*, nominal *p* value; q, FDR-adjusted *p* value within each grouping-variable family across the 12 analytes.

**Table 3:** Primary adjusted regression models for each human milk hormone and cytokine.

Panel A. Regression coefficients from the primary adjusted models							
Analyte (*n*)	Model Term	β (log [pg/mL + 1])	SE	Model *p* value	95% CI		q
Ghrelin (*n* = 39)	Infant age	0.004	0.044	0.930	−0.086 to 0.093		0.930
Ghrelin (*n* = 39)	Postpartum Maternal BMI	0.013	0.026	0.632	−0.040 to 0.065		0.718
Ghrelin (*n* = 39)	Infant sex: male vs. female	0.121	0.183	0.512	−0.250 to 0.492		0.954
Ghrelin (*n* = 39)	Birth mode	−0.207	0.179	0.254	−0.571 to 0.156		0.674
Glucagon (*n* = 37)	Infant age	−0.004	0.036	0.922	−0.078 to 0.071		0.930
Glucagon (*n* = 37)	Postpartum Maternal BMI	0.031	0.022	0.174	−0.014 to 0.076		0.450
Glucagon (*n* = 37)	Infant sex: male vs. female	−0.156	0.157	0.329	−0.476 to 0.165		0.954
Glucagon (*n* = 37)	Birth mode	−0.579	0.153	<0.001	−0.890 to −0.268		0.007
Insulin (*n* = 37)	Infant age	0.038	0.069	0.581	−0.102 to 0.178		0.881
Insulin (*n* = 37)	Postpartum Maternal BMI	−0.002	0.040	0.959	−0.083 to 0.079		0.959
Insulin (*n* = 37)	Infant sex: male vs. female	−0.000	0.266	0.999	−0.543 to 0.542		0.999
Insulin (*n* = 37)	Birth mode	0.162	0.275	0.559	−0.398 to 0.722		0.746
Leptin (*n* = 39)	Infant age	−0.009	0.069	0.899	−0.150 to 0.132		0.930
Leptin (*n* = 39)	Postpartum Maternal BMI	0.152	0.041	<0.001	0.068 to 0.236		0.010
Leptin (*n* = 39)	Infant sex: male vs. female	0.176	0.314	0.579	−0.463 to 0.815		0.954
Leptin (*n* = 39)	Birth mode	−0.243	0.312	0.442	−0.878 to 0.392		0.674
IFN-γ (*n* = 39)	Infant age	0.149	0.072	0.047	0.002 to 0.296		0.363
IFN-γ (*n* = 39)	Postpartum Maternal BMI	0.070	0.042	0.104	−0.015 to 0.156		0.450
IFN-γ (*n* = 39)	Infant sex: male vs. female	−0.096	0.301	0.752	−0.707 to 0.515		0.954
IFN-γ (*n* = 39)	Birth mode	−0.236	0.308	0.449	−0.863 to 0.391		0.674
IL-1β (*n* = 44)	Infant age	0.062	0.056	0.271	−0.050 to 0.175		0.881
IL-1β (*n* = 44)	Postpartum Maternal BMI	0.027	0.033	0.410	−0.039 to 0.093		0.703
IL-1β (*n* = 44)	Infant sex: male vs. female	0.126	0.244	0.609	−0.367 to 0.618		0.954
IL-1β (*n* = 44)	Birth mode	0.051	0.233	0.829	−0.420 to 0.522		0.904
IL-2 (*n* = 39)	Infant age	0.038	0.060	0.533	−0.085 to 0.161		0.881
IL-2 (*n* = 39)	Postpartum Maternal BMI	0.043	0.035	0.225	−0.028 to 0.115		0.450
IL-2 (*n* = 39)	Infant sex: male vs. female	0.097	0.251	0.703	−0.414 to 0.608		0.954
IL-2 (*n* = 39)	Birth mode	−0.230	0.258	0.379	−0.754 to 0.294		0.674
IL-4 (*n* = 38)	Infant age	−0.075	0.081	0.362	−0.239 to 0.090		0.881
IL-4 (*n* = 38)	Postpartum Maternal BMI	0.059	0.047	0.213	−0.036 to 0.154		0.450
IL-4 (*n* = 38)	Infant sex: male vs. female	0.422	0.347	0.232	−0.283 to 1.128		0.954
IL-4 (*n* = 38)	Birth mode	−0.005	0.352	0.988	−0.721 to 0.710		0.988
IL-6 (*n* = 41)	Infant age	0.032	0.072	0.661	−0.115 to 0.179		0.881
IL-6 (*n* = 41)	Postpartum Maternal BMI	0.025	0.042	0.544	−0.059 to 0.110		0.718
IL-6 (*n* = 41)	Infant sex: male vs. female	0.459	0.321	0.161	−0.191 to 1.109		0.954
IL-6 (*n* = 41)	Birth mode	−0.533	0.307	0.091	−1.156 to 0.089		0.544
IL-7 (*n* = 44)	Infant age	0.029	0.059	0.629	−0.091 to 0.149		0.881
IL-7 (*n* = 44)	Postpartum Maternal BMI	0.048	0.036	0.190	−0.025 to 0.120		0.450
IL-7 (*n* = 44)	Infant sex: male vs. female	0.049	0.261	0.853	−0.478 to 0.576		0.954
IL-7 (*n* = 44)	Birth mode	−0.215	0.247	0.388	−0.714 to 0.283		0.674
MCP-1 (*n* = 44)	Infant age	−0.221	0.114	0.061	−0.452 to 0.010		0.363
MCP-1 (*n* = 44)	Postpartum Maternal BMI	0.042	0.070	0.557	−0.101 to 0.184		0.718
MCP-1 (*n* = 44)	Infant sex: male vs. female sex	−0.174	0.499	0.730	−1.183 to 0.836		0.954
MCP-1 (*n* = 44)	Birth mode	0.214	0.476	0.656	−0.750 to 1.178		0.787
TNF-α (*n* = 44)	Infant age	0.042	0.054	0.443	−0.068 to 0.152		0.881
TNF-α (*n* = 44)	Postpartum Maternal BMI	−0.014	0.032	0.658	−0.078 to 0.050		0.718
TNF-α (*n* = 44)	Infant sex: male vs. female	0.037	0.233	0.875	−0.435 to 0.509		0.954
TNF-α (*n* = 44)	Birth mode	−0.179	0.225	0.430	−0.634 to 0.275		0.674
Panel B. Model diagnostics for the primary adjusted models							
Analyte	*n*	Shapiro–Wilk *p*	Breusch–Pagan *p*	Maximum VIF		Influential observations	
Ghrelin	39	0.101	0.146	1.24		1	
Glucagon	37	0.792	0.033	1.20		2	
Insulin	37	0.540	0.082	1.23		1	
Leptin	39	0.256	0.203	1.28		1	
IFN-γ	39	0.038	0.638	1.15		2	
IL-1β	44	0.470	0.859	1.13		4	
IL-2	39	0.024	0.153	1.15		1	
IL-4	38	0.007	0.219	1.16		2	
IL-6	41	0.208	0.536	1.15		2	
IL-7	44	0.261	0.537	1.09		3	
MCP-1	44	0.039	0.415	1.10		1	
TNF-α	44	0.281	0.103	1.14		3	

Milk hormone or cytokine concentrations were modeled as dependent variables after log(concentration + 1) transformation. Each primary model included infant age at recruitment, postpartum maternal BMI, infant sex and birth mode simultaneously. Panel A reports the regression coefficients from these primary models. All model terms are shown, regardless of statistical significance. β values are regression coefficients on the log(concentration + 1) scale. Infant age was entered in months and postpartum maternal BMI in kg/m^2^. Female infants and vaginal delivery were used as reference categories for infant sex and birth mode, respectively. In Panel A, *p* values are nominal, and q values are FDR-adjusted. Panel B reports model diagnostics. Influential observations were defined by Cook’s distance > 4/*n*. Abbreviations: BMI, body mass index; SE, standard error; CI, confidence interval; FDR, false-discovery rate; VIF, variance inflation factor.

## Data Availability

The data presented in this study are available from the corresponding author upon reasonable request. The data are not publicly available due to privacy and ethical restrictions.

## Abbreviations

The following abbreviations are used in this manuscript:

BAZ	BMI-for-age Z-score
BMI	body mass index
CD	Cesarean delivery
HCZ	Head-circumference-for-age Z-score
LAZ	Length-for-age Z-score
VD	Vaginal delivery
WAZ	Weight-for-age Z-score
WHO	World Health Organization
WLZ	Weight-for-length Z-score
